# MicroRNA Expression in High‐Grade B‐Cell Lymphoma With 11q Aberration

**DOI:** 10.1002/gcc.70021

**Published:** 2025-01-23

**Authors:** Gioia Di Stefano, Anja Fischer, Emil Chteinberg, Susanne Bens, Rabea Wagener, Dmitriy Abramov, Patrick Adam, Stephan H. Bernhart, Arndt Borkhardt, Birgit Burkhardt, Coral Del‐Val, Michael C. Frühwald, Raffaella Guazzo, Jessica I. Hoell, Michael Hummel, Heike Horn, Wolfram Klapper, Jens Krugmann, Katrin S. Kurz, Stefano Lazzi, Abner Jr. Louissaint, Anja Mottok, Ilske Oschlies, Raffaella Santi, Kristian Schafernak, Annette M. Staiger, Yanming Zhang, Andreas Rosenwald, Lorenz Trümper, Lorenzo Leoncini, German Ott, Reiner Siebert

**Affiliations:** ^1^ Histopathology and Molecular Diagnostics Careggi University Hospital Florence Italy; ^2^ Institute of Human Genetics Ulm University and Ulm University Medical Center Ulm Germany; ^3^ Department of Pathology, Dmitry Rogachev National Medical Research Center of Pediatric Hematology Oncology and Immunology Moscow Russia; ^4^ Pathologie Ingolstadt and Institute of Pathology, TUM School of Medicine and Health Technical University Munich (TUM) Munich Germany; ^5^ Bioinformatics Group, Department of Computer Science and Interdisciplinary Center for Bioinformatics Leipzig University Leipzig Germany; ^6^ Department of Pediatric Oncology, Haematology and Clinical Immunology, Medical Faculty Heinrich‐Heine‐University Düsseldorf Germany; ^7^ Pediatric Hematology and Oncology University Hospital Muenster Muenster Germany; ^8^ Department of Computer Science and Artificial Intelligence, University of Granada Andalusian Research Institute in Data Science and Computational Intelligence Granada Spain; ^9^ Instituto de Investigación Biosanitaria Ibs. Granada Granada Spain; ^10^ Pediatrics and Adolescent Medicine, Swabian Children's Cancer Center University Hospital Augsburg Augsburg Germany; ^11^ Section of Pathology, Department of Medical Biotechnologies University of Siena Siena Italy; ^12^ Paediatric Haematology and Oncology Martin Luther University Halle‐Wittenberg Halle (Saale) Germany; ^13^ Institute of Pathology, Charité‐Universitätsmedizin Berlin, Corporate Member of Freie Universität Berlin Humboldt‐Universität Zu Berlin & Berlin Institute of Health Berlin Germany; ^14^ Department of Clinical Pathology Robert‐Bosch‐Krankenhaus Stuttgart Germany; ^15^ Dr. Margarete Fischer‐Bosch Institute of Clinical Pharmacology, Stuttgart, and University of Tübingen Germany; ^16^ Section Haematopathology and Lymph Node Registry, Department of Pathology Christian‐Albrechts‐University Kiel Kiel Germany; ^17^ Institut für Pathologie Klinikum Bayreuth Bayreuth Germany; ^18^ Department of Pathology Massachusetts General Hospital Boston Massachusetts USA; ^19^ Department of Pathology University of Würzburg Würzburg Germany; ^20^ Pathology Section, Department of Health Sciences University of Florence Florence Italy; ^21^ Division of Pathology and Laboratory Medicine Phoenix Children's Hospital Phoenix Arizona USA; ^22^ Department of Pathology and Laboratory Medicine Memorial Sloan Kettering Cancer Center New York New York USA; ^23^ Department of Hematology and Oncology Georg‐August University Göttingen Göttingen Germany

**Keywords:** Burkitt lymphoma, diffuse large B‐cell lymphoma, high‐grade B‐cell lymphoma with 11q aberration, micro‐RNAs

## Abstract

Mature aggressive B‐cell lymphomas, such as Burkitt lymphoma (BL) and Diffuse large B‐cell lymphoma (DLBCL), show variations in microRNA (miRNA) expression. The entity of High‐grade B‐cell lymphoma with 11q aberration (HGBCL‐11q) shares several biological features with both BL and DLBCL but data on its miRNA expression profile are yet scarce. Hence, this study aims to analyze the potential differences in miRNA expression of HGBCL‐11q compared to BL and DLBCL. We evaluated the expression profiles of 2083 miRNAs in 25 HGCBL‐11q, 7 BL, 131 DLBCL, and tonsils using the HTG EdgeSeq miRNA whole transcriptome assay. Uniform manifold approximation and projection (UMAP) and differential gene expression analyses based on DESeq2 were carried out. UMAP analysis of miRNA expression did not reveal distinct groups among the studied lymphomas. However, differential gene expression investigations detected sets of overexpressed miRNAs in HGBCL‐11q when compared to BL (miR‐9‐3p, miR‐9‐5p, miR‐3919, miR‐129‐1‐3p, miR‐129‐2‐3p, miR‐331‐3p, miR‐196b‐5p, and miR‐28‐5p) and DLBCL (miR‐3919, miR‐1290, miR‐4538, and miR‐4791), respectively. Notably, miR‐3919 showed heterogeneous but significantly higher expression (*p*‐value < 0.001) in HGBCL‐11q than in both, BL and DLBCL. We identified a group of differentially expressed miRNAs between HGBCL‐11q vs. BL and DLBCL, with miR‐3919 as the most commonly and recurrently overexpressed miRNA in HGBCL‐11q.

**Abbreviations**
BL: Burkitt lymphoma
DE miRNAs: differentially expressed miRNAs
DLBCL: diffuse large B‐cell lymphoma
HGBCL‐11q: high‐grade lymphoma with 11q aberration
ICGC: International Cancer Genome Consortium
MMML‐Seq: molecular mechanisms in malignant lymphoma by sequencing
QC: quality control

## 1 Introduction

MicroRNAs (miRNAs) are small, non‐coding, single‐stranded RNA molecules that regulate physiological processes and malignant transformation in B‐cells at a post‐transcriptional level [1]. In mature B‐cell lymphomas, a group of miRNAs helped to distinguish between different categories, such as Diffuse large B‐cell lymphoma (DLBCL) and Follicular lymphoma (FL) [2], but also, miRNA profiling was able to discriminate between the DLBCL germinal center B‐cell (GCB) and activated B‐cell (ABC) subtypes [3]. Additionally, a fair amount of MYC‐regulated and nuclear factor‐kappa B pathway associated miRNAs was useful in differentiating between Burkitt lymphoma (BL) and DLBCL [4, 5].

High‐grade B‐cell lymphoma with 11q aberration (HGBCL‐11q) is a mature aggressive B‐cell lymphoma that shares some histopathological and molecular aspects with both BL and DLBCL [6, 7]. In particular, HGBCL‐11q shows morphological and gene expression features of BL whereas its mutational profile and the pattern of structural variants resembles that of DLBCL [7, 8]. With these characteristics, the current WHO classification (WHO‐HAEM5) accepts it as a distinct aggressive B‐cell lymphoma entity [9]. While HGBCL‐11q mRNA expression has been explored since its original description [6], to the best of our knowledge still no data on miRNA expression in HGBCL‐11q have been published. Therefore, we herein investigated the miRNA expression profile in HGBCL‐11q and compared it to BL as well as DLBCL.

## 2 Materials and Methods

### 2.1 Study Cohort

MiRNA gene expression analysis was conducted on formalin‐fixed, paraffin‐embedded (FFPE) tissue of 25 HGBCL‐11q, seven BL, and 131 DLBCL, along with two tonsil specimens (with 5 and 6 replicates for tonsil 1 and 2, respectively). The BL were retrieved from previous studies [10] while the DLBCLs belong to several clinical trials conducted by the German High‐Grade Non‐Hodgkin's Lymphoma Study Group, including RICOVER‐60 [11] (*n* = 11), RICOVER‐noRTh [12] (*n* = 27), DENSE‐R [13] (*n* = 18), MegaCHOEP [14] (*n* = 17), MegaCHOEP III (*n* = 13) [14], SMARTE‐R [15] (*n* = 27), and MInT [16] (*n* = 18). These trials follow the Helsinki declaration, and the protocols have been approved by the respective ethics review committee of each participating center. Of the 25 HGBCL‐11q, seven have been analyzed in previously published studies [6, 7, 17, 18]. The remaining 18 HGBCL‐11q cases were obtained from multiple institutions and study groups and met the classification criteria outlined in the 5th edition of the World Health Organization following histopathological and cytogenetic investigations using fluorescence in situ hybridization (FISH) and/or OncoScan technology (Thermo Fisher Scientific, Waltham, MA, USA). Moreover, sequencing data of small RNAs from the ICGC (International Cancer Genome Consortium)‐MMML‐Seq (Molecular Mechanisms in Malignant Lymphoma by Sequencing) (ICGC MMML‐Seq) project were mined [5]. The study followed the guidelines of ICGC MMML‐Seq approved by the Medical Faculty's Ethical Committee at University of Kiel (A150/10) and Ulm University (349/11), and other institutions involved.

### 2.2 Expression Profiling by HTG EdgeSeq Assays

In this study, we evaluated the expression profiles of 2083 miRNAs through the “HTG EdgeSeq miRNA whole transcriptome assay” according to the manufacturer's instructions (HTG Molecular Diagnostics, Tucson, USA). Briefly, FFPE samples (a minimum of 6 mm^2^ tissue in 10 μm thick sections) were lysed by heating in lysis buffer at 95°C for 20 min and subsequently incubated with Proteinase K at 50°C for 3 h. After performing the quantitative nuclease protection step in the HTG EdgeSeq System, the resulting probes were amplified, purified using AMPure XP beads (Beckman Coulter, Brea, CA, USA), and analyzed by quantitative PCR using the KAPA Library Quantification Kit (KAPA Biosystems, Wilmington, MA, USA) according to the manufacturer's instructions. The concentration in each library was calculated using the HTG RUO Library Calculator. The pooled equimolar libraries were diluted and loaded onto an Illumina NextSeq 550 instrument, according to the manufacturer's recommendations (Illumina, San Diego, USA). Read quantification from Fastq files was performed using HTG EdgeSeq parser software, and quality control was performed using HTG Reveal software version 4.0.1.

Moreover, the cell of origin (COO) signature of all samples was assessed using transcriptional profiling with the HTG EdgeSeq Pan B‐Cell Lymphoma Panel (HTG Molecular Diagnostics, Tucson, USA) followed by application of the DLBCL automatic classification (DAC) algorithm [19].

### 2.3 Dimension Reduction and Differential Gene Expression Analysis

Uniform manifold approximation and projection (UMAP) was performed on the log2 transformed gene expression values (CPM values) using the umap package (version 0.2.10.0) with default and different parameters (number of neighbors 5–20, distance: Euclidean and Manhattan) and visualized using the ggplot2 package (3). Differential gene expression analysis was performed on raw counts using the DESeq2 package (version 1.42.1). Data were adjusted using adaptive shrinkage in a high‐dimensional function (ashr). The obtained *p*‐values were corrected for multiple testing using Benjamini‐Hochberg. In addition, the Linear Models for Microarray Data (limma) package (3.56.2) was used as the second platform to estimate differentially expressed genes. The limma “lmFit” function was handled to fit a linear model to the gene expression data, with the design matrix as the input. The “eBayes” function was placed to compute moderated t‐statistics and log2 fold changes. Differential gene expression was considered significant when the adjusted *p*‐value was less than 0.05, and the log2 fold change was less than −1 or greater than 1. The ggplot2 package was applied to generate volcano plots. The log2 fold change is plotted on the *x*‐axis, and the negative log10 of the adjusted *p*‐value is plotted on the *y*‐axis. The pheatmap package (version1.0.12) was employed to generate heatmaps of the significantly differently expressed miRNAs (DE miRNAs). All computational analyses were performed in R (version 4.3.1).

## 3 Results

### 3.1 Quality Control and UMAP Analysis Based on miRNA Assay

**FIGURE 1:**
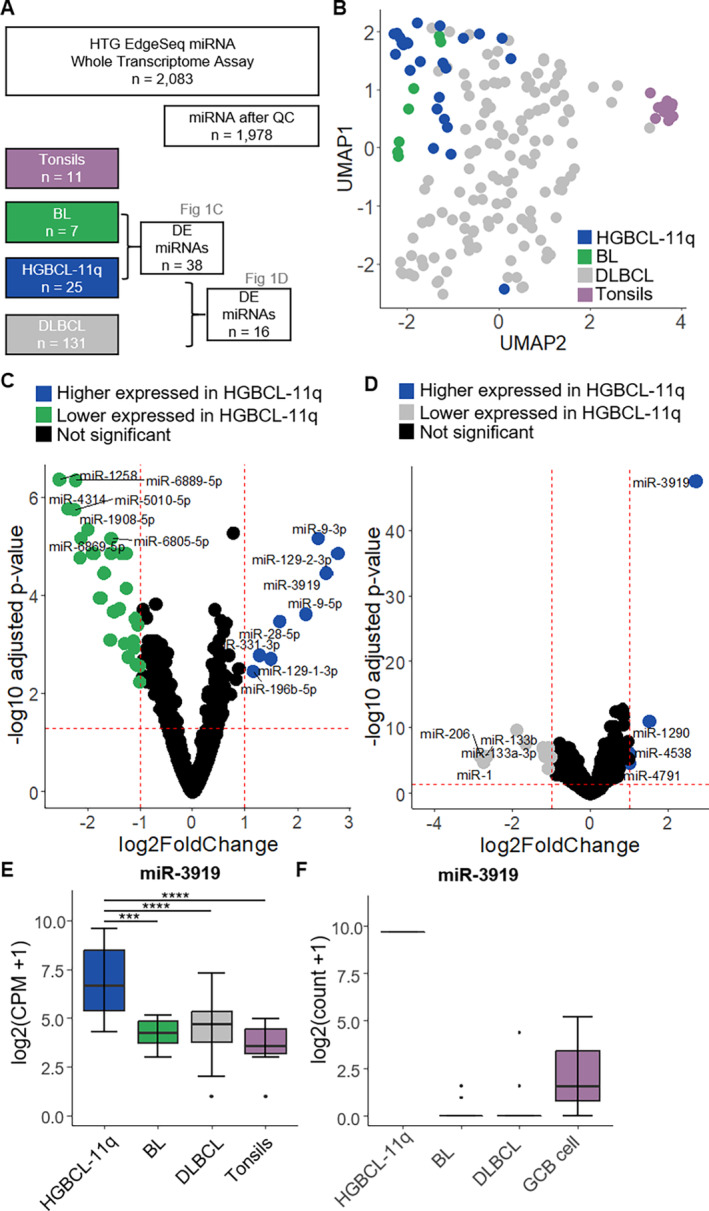
MiRNA expression in HGBCL‐11q compared to BL and DLBCL. A: Overview of study cohort. Number of samples per group and miRNAs are indicated before and after QC with also the DE miRNAs. B: Uniform Manifold Approximation and Projection (UMAP, default parameters) based on the miRNA expression (*n* = 1978) of HGBCL‐11q (*n* = 25), BL (*n* = 7) and DLBCL (*n* = 131) and tonsillar controls (*n* = 11, two tonsils with 5 and 6 replicates). C: The volcano plot displays the DE miRNAs between HGBCL‐11q and BL using DESeq2. The DE miRNAs are colored according to their log2 fold change and adjusted *p*‐value, and the horizontal and vertical lines represent significance thresholds. Please note the different axis scaling in C and D. D: The volcano plot displays the DE miRNAs between HGBCL‐11q and DLBCL using DESeq2. The DE miRNAs are colored according to their log2 fold change and adjusted *p*‐value, and the horizontal and vertical lines represent significance thresholds. Please note the different axis scaling in C and D. E: Expression of miR‐3919 within the mature aggressive B‐cell lymphoma entities in this study: HGBCL‐11q (*n* = 25), BL (*n* = 7), DLBCL (*n* = 131) and tonsillar controls (*n* = 11, two tonsils with 5 and 6 replicates). Pairwise, two‐sided Wilcoxon test with Benjamini‐Hochberg correction for multiple testing. ***p < 0.001, ****p < 0.0001. Boxplot settings: Middle, median; lower hinge, 25% quantile; upper hinge, 75% quantile; upper/lower whisker, largest/smallest observation less/greater than or equal to upper/lower hinge ±1.5 * IQR. F: MiR‐3919 expression in HGBCL (HGBCL‐11q [*n* = 1], BL [*n* = 15], DLBCL [*n* = 11] and germinal center B cell populations [GCB cells, *n* = 3]) determined by small RNAseq within the ICGC MMML‐Seq project [7]. Boxplot settings: Middle, median; lower hinge, 25% quantile; upper hinge, 75% quantile; upper/lower whisker, largest/smallest observation less/greater than or equal to upper/lower hinge ±1.5 * IQR.

Our study examined the expression of 2083 miRNAs in 25 HGBCL‐11q, 7 BL, and 131 DLBCL using the HTG EdgeSeq miRNA whole transcriptome assay. All samples met the quality criteria recommended by the supplier (Figure S1). Nevertheless, for the differential expression analysis we excluded a set of 105 miRNAs that showed low mean expression (< 1.6 of log2 transformed quantile normalized counts plus one (QNC + 1)) across all samples, leading to a total of 1978 miRNAs entering the downstream analyses (Figure 1A). Visualization of the expression of these 1978 miRNAs using UMAP separated the tonsillar control tissues from the mature aggressive B‐cell lymphomas (Figure 1B). On the other hand, although UMAP provided some structure to the cases, it did not form distinct clusters for the lymphoma subtypes. Therefore, the unsupervised analyses of the expression values of 1978 miRNAs could not unambiguously separate the lymphoma entities from each other.

### 3.2 Differentially Expressed miRNAs in HGBCL‐11q Compared to BL and DLBCL

We used DESeq2 analysis to identify miRNAs expressed differentially between BL and HGBCL‐11q. We considered miRNAs with an adjusted *p* value < 0.05 and a log2 fold change less than −1 or greater than 1 as significantly differentially expressed (Figure 1C). As a result, we found 38 differentially expressed miRNAs in HGBCL‐11q compared to BL, with 8 higher and 30 lower expressed. In particular, the miRNAs higher expressed in HGBCL‐11q were miR‐9‐3p, miR‐9‐5p, miR‐3919, miR‐129‐2‐3p, miR‐129‐1‐3p, miR‐331‐3p, miR‐196b‐5p, and miR‐28‐5p. Instead, the miRNAs lower expressed in HGBCL‐11q compared to BL are, among others, miR‐4314, miR‐1258, miR‐6889‐5p, miR‐6869‐5p, miR‐6805‐5p, miR‐1908‐5p, and miR‐5010‐5p. Therefore, from the analysis performed, we identified a group of miRNAs that were significantly higher expressed in HGBCL‐11q compared to BL. Notably, most of these miRNAs showed a pronounced variable expression within the HGBCL‐11q group, as shown in the corresponding heatmap (Figure S2).

The analysis using DESeq2 to compare miRNA expression between HGBCL‐11q and DLBCL revealed 16 significantly differentially expressed miRNAs. Four miRNAs were higher and 12 were lower expressed in HGBCL‐11q. The higher expressed miRNAs in HGBCL‐11q were miR‐3919, miR‐1290, miR‐4538, and miR‐4791, while the lower expressed miRNAs were, among others, miR‐133a‐3p, miR‐206, miR‐133b, and miR‐1 compared to DLBCL. The heatmap showed that the differential expression of these miRNAs can distinguish separate groups of HGBCL‐11q, and again indicates a heterogeneous expression within HGBCL‐11q (Figure S2). Whereas DLBCL includes GCB, ABC, and unclassified subtypes, HGBCL‐11q usually displays a GCB expression signature. To rule out that the different COO subtypes in DLBCL affect differential expression analysis to HGBCL‐11q, we next specifically focused on comparing HGBCL‐11q to only the DLBCL‐GCB samples (*n* = 49). Our analysis revealed 18 differentially expressed miRNAs (4 higher and 14 lower expressed in HGBCL‐11q). No differences in the higher expressed miRNAs compared to the previous analysis (miR‐3919, miR‐1290, miR‐4538, and miR‐4791) were identified. However, the group of miRNAs expressed at lower levels in HGBCL‐11q showed some variations. For instance, miR‐135b‐5p, miR‐146b‐5p, miR‐222‐5p, and miR‐155‐3p (Figure S2) were uniquely lower expressed in HGBCL‐11q compared to DLBCL, while miR‐3681‐5p, miR‐208b‐3p, miR‐146a‐5p, miR‐129‐1‐3p, miR‐129‐2‐3p and miR‐4524a‐5p were only identified in the comparison between HGBCL‐11q and DLBCL‐GCB (Figure S3). Of note, miR‐129‐2‐3p was higher expressed in HGBCL‐11q compared to BL, but as shown from this latter result it is lower expressed in HGBCL‐11q vs. DLBCL‐GCB. Furthermore, a DESeq2 analysis was performed to find the miRNAs differentially expressed between BL and DLBCL, thus revealing several downregulated (miR‐664a‐3p, miR‐155‐5p, miR‐196b‐5p) and upregulated miRNAs (miR‐25‐5p and miR‐18a‐3p) in BL compared to DLBCL which have resulted also in distinguishing both entities in previous publications [5].

### 3.3 MiR‐3919 is the Only miRNA Differentially Expressed in HGBCL‐11q Compared to BL and DLBCL

In HGBCL‐11q, miR‐3919 showed a particularly higher expression in DESeq2 analysis compared to both, DLBCL and BL, respectively. While there is some variation in the expression of miR‐3919 within the HGBCL‐11q group, it was significantly higher expressed in HGBCL‐11q compared to BL, DLBCL and the tonsils (Figure 1E). To validate this finding with an orthogonal method, we also examined miR‐3919 expression in the small RNA sequencing dataset of the ICGC MMML‐Seq cohort [5]. Indeed, we found that compared to BL, DLBCL and normal GCB cells, the highest expression of miR‐3919 was in the only HGBCL‐11q sample (4135350) included in the ICGC MMML‐Seq small RNA sequencing dataset but which was not analyzed herein (Figure 1F). Therefore, out of all the miRNAs detected through the differential miRNA expression analysis, miR‐3919 seems to be most consistently overexpressed in HGBCL‐11q, implying its potential relevance in at least a subset of cases of this type of lymphoma.

## 4 Discussion

A set of miRNAs demonstrated a consistent expression pattern in aggressive B‐cell lymphomas such as BL [4] and DLBCL [5]. This indicates the possibility of distinguishing between these types of tumors based on miRNA profiling as well as illustrating a potential biological impact in the pathogenesis of these malignancies. Conversely, the miRNA expression in HGBCL‐11q has yet to be examined and to address this gap, we compared the miRNA expression in HGBCL‐11q to BL and DLBCL. Although unsupervised UMAP analysis did not show a clear separation between the lymphoma entities interrogated, further investigation of the differential miRNA expression revealed a group of significantly differentially expressed miRNA in HGBCL‐11q compared to the other two categories (BL and DLBCL) thus suggesting that HGBCL‐11q has a distinct miRNA expression signature, albeit with heterogeneous expression levels. A group of miRNAs display significantly higher expression levels and most of them (i.e., miR‐9, miR‐28‐5p, miR‐129‐2‐3, and miR‐331‐3p) are typically present in normal GC B‐cells, specifically in centroblasts [20, 21]. Although previous studies on non‐neoplastic B‐cell miRNAs indicated that the regular expression pattern of B‐cell lineage‐specific miRNAs is maintained in the malignant counterpart [20] our DESeq2 analysis highlighted a distinct difference in the GC‐expressed miRNAs in HGBCL‐11q when compared to other mature GCB lymphomas. For example, miR‐9, which has been reported to be highly expressed in BL compared to DLBCL [5], is much more overexpressed in HGBCL‐11q than in BL. MiR‐28‐5p which was previously described as lower expressed in BL [22] was instead higher expressed in HGBCL‐11q compared to BL. Additionally, miR‐129‐2‐3p is overexpressed in HGBCL‐11q than BL but lower expressed when compared to DLBCL‐GCB. This latter miRNA has been reported to be lower expressed in BL whereas it was overexpressed in a subset of DLBCL compared to FL [5].

HGBCL‐11q and DLBCL showed differential expression of specific miRNAs, with variability observed when comparing HGBCL‐11q to DLBCL or DLBCL‐GCB, specifically for the lower expressed miRNAs. For instance, miR‐222 was found to be significantly lower expressed in HGBCL‐11q compared to all DLBCL but not compared to DLBCL‐GCB. It was also reported to be expressed at lower levels in BL vs. DLBCL [4]. Furthermore, previous studies have indicated that miR‐222 is also lower expressed in normal GC B‐cells compared to plasma cells, memory B‐cells [23] and naive B‐cells [20].

Among the various highlighted miRNAs, just one miRNA, miR‐3919, was consistently higher expressed in HGBCL‐11q in all DESeq2 analyses, making it the most intriguing and unexplored. It is worth mentioning that miR‐3919 by an alternative bioinformatics approach using Limma analysis did not show significant expression in the HGBCL‐11q vs. BL comparison, most likely due to its heterogeneous expression in HGBCL‐11q or sample size. Moreover, in previous publications miR‐3919 was reported to be expressed in the EBV‐positive BL lymphoma cell line Daudi [23]. However, miR‐3919 was highly expressed exclusively in the HGBCL‐11q group and in most of the samples (60%). In fact, miR‐3919 showed high expression in the single HGBCL‐11q case from the ICGC MMML‐Seq cohort that was tested by an alternative technology, supporting its expression to be associated with HGBCL‐11q. Regarding the function of miR‐3919, despite it being rarely reported, recent research has found that miR‐3919 is among a cluster of miRNAs that seems to be moderately increasing with age [24], although in the HGBCL‐11q groups we did not observe a correlation between the miR‐3919 expression and age difference; also, miR‐3919 appears to play a pathogenic role in certain epithelial tumors, such as prostate cancer [25]. Additionally, its expression has been identified in aggressive mature B‐cell tumors [23], and based on our results, especially in HGBCL‐11q. However, its particular monitoring task in this context is still unknown. Further investigations are needed to better understand the role of miR‐3919. However, its presence in FFPE samples may serve as a potential biomarker for diagnosing HGBCL‐11q. This opportunity is attributed to its significant expression levels and the observation that miRNAs are generally better preserved than mRNA during formalin fixation and paraffin embedding. Additionally, detecting miR‐3919 through liquid biopsy could extend its clinical utility. In conclusion, we unraveled a differential miRNA expression profile between HGBCL and detected a group of differentially expressed miRNAs in HGBCL‐11q compared to BL and DLBCL, with miR‐3919 being the most recurrently overexpressed miRNA in HGBCL‐11q.

## Conflicts of Interest

Reiner Siebert: Our laboratory received reagents for reduced prices from HTG for testing the technology. All other authors declare no conflicts of interest.

## Supporting information


**Figure S1.** Quality control (QC) assessment. The three HTG quality steps are shown (QC0–QC2). All samples passed the control evaluation. QC measurements are shown for HGBCL‐11q (*n* = 25), BL (*n* = 7), DLBCL (*n* = 131) and tonsil samples (*n* = 11, two tonsils with 5 and 6 replicates).

**Figure S2.** Heatmap displaying the differential miRNA expression between HGBCL‐11q, BL and DLBCL. The heatmap illustrates the relative expression levels of DE miRNAs. Rows represent miRNAs and columns display the samples, with a color gradient from blue (low expression) to red (high expression) indicating the z‐score normalized expression values. Hierarchical clustering was performed for both miRNAs and samples, revealing distinct expression patterns and sample grouping. A: The heatmap representing the miRNA expression of HGBCL‐11q vs. BL. B: The heatmap representing the miRNA expression of HGBCL‐11q vs. DLBCL.

**Figure S3.** Differential miRNA expression between HGBCL‐11q and DLBCL, GCB. A: The volcano plot displays the DE miRNAs between HGBCL‐11q and DLBCL‐GCB using DESseq2. The DE miRNAs are colored according to their log2 fold change and adjusted *p*‐value, and the horizontal and vertical lines represent significance thresholds. B: The heatmap illustrates the relative expression levels of DE miRNAs. Rows represent miRNAs and columns show the samples, with a color gradient from blue (low expression) to red (high expression) indicating the z‐score normalized expression values. Hierarchical clustering was performed for both miRNAs and samples, revealing distinct expression patterns and sample grouping.

## Data Availability

Raw count data is available in Supplementary data. There is an excel file that was submitted with the following name.
