# Supplementary figures and images for: MicroRNA Expression in High‐Grade B‐Cell Lymphoma With 11q Aberration
Source: Genes Chromosomes Cancer. 2025 Jan 23;64(1):e70021. doi: 10.1002/gcc.70021 (PMC11755218; doi:10.1002/gcc.70021)

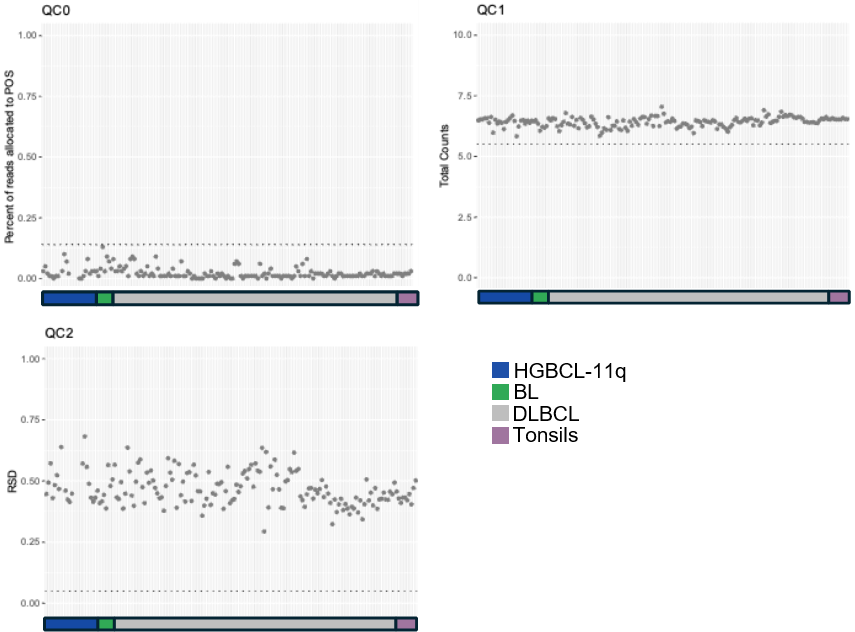

Supplement: Supplementary file 1 — Figure S1. Quality control (QC) assessment. The three HTG quality steps are shown (QC0–QC2). All samples passed the control evaluation. QC measurements are shown for HGBCL‐11q (n = 25), BL (n = 7), DLBCL (n = 131) and tonsil samples (n = 11, two tonsils with 5 and 6 replicates). [file GCC-64-e70021-s003.tif]

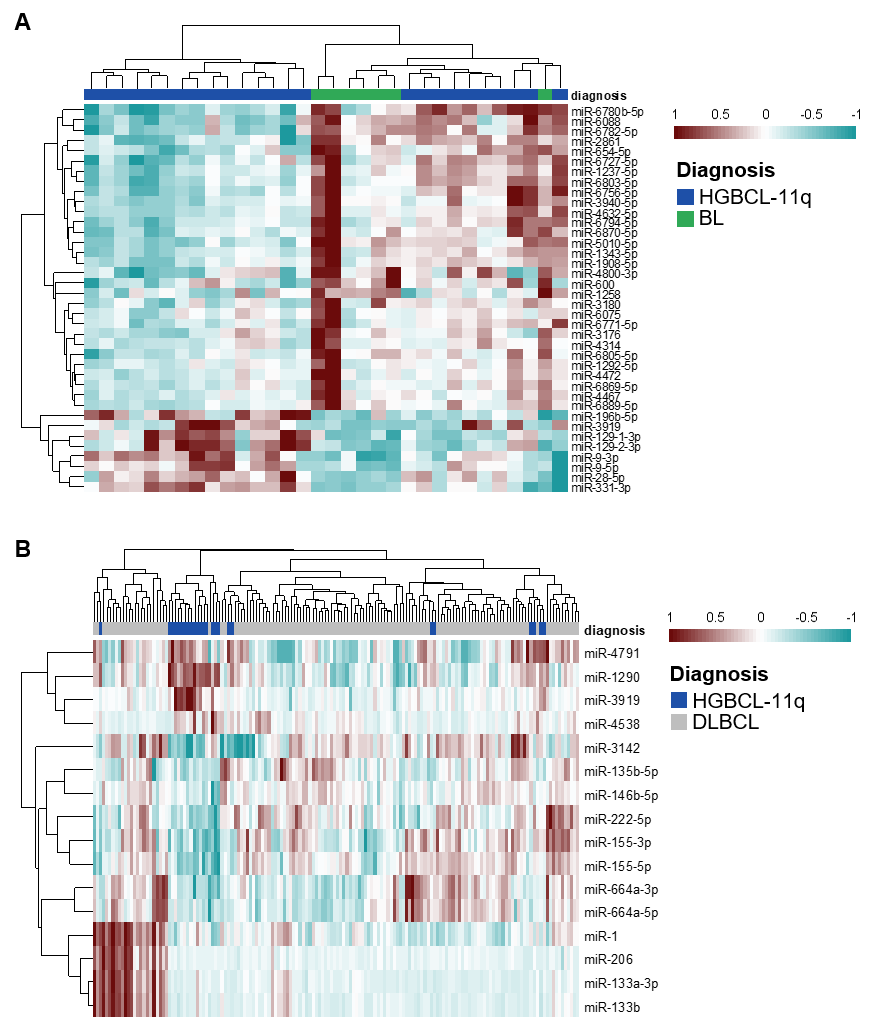

Supplement: Supplementary file 2 — Figure S2. Heatmap displaying the differential miRNA expression between HGBCL‐11q, BL and DLBCL. The heatmap illustrates the relative expression levels of DE miRNAs. Rows represent miRNAs and columns display the samples, with a color gradient from blue (low expression) to red (high expression) indicating the z‐score normalized expression values. Hierarchical clustering was performed for both miRNAs and samples, revealing distinct expression patterns and sample grouping. A: The heatmap representing the miRNA expression of HGBCL‐11q vs. BL. B: The heatmap representing the miRNA expression of HGBCL‐11q vs. DLBCL. [file GCC-64-e70021-s001.tif]

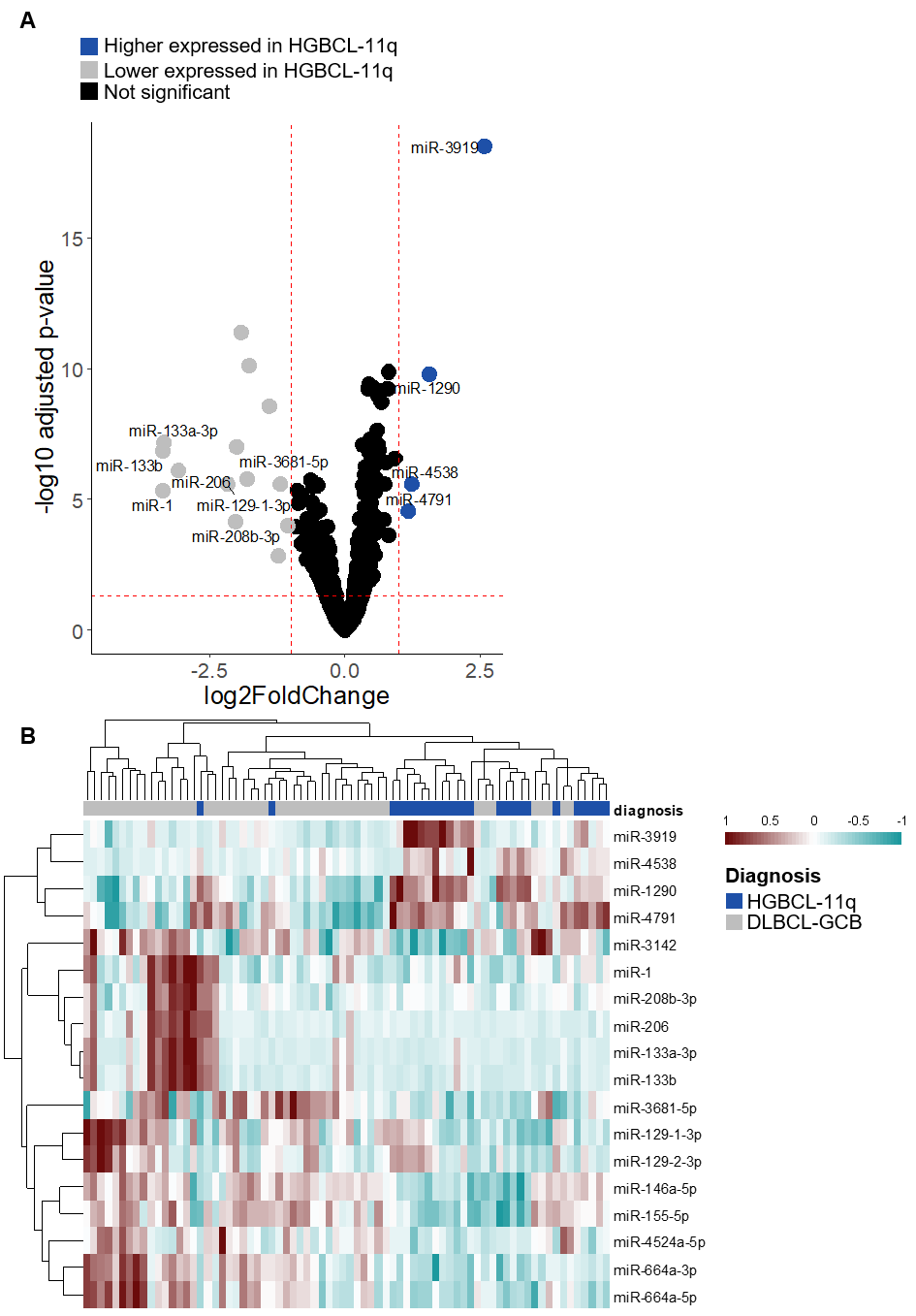

Supplement: Supplementary file 3 — Figure S3. Differential miRNA expression between HGBCL‐11q and DLBCL, GCB. A: The volcano plot displays the DE miRNAs between HGBCL‐11q and DLBCL‐GCB using DESseq2. The DE miRNAs are colored according to their log2 fold change and adjusted p‐value, and the horizontal and vertical lines represent significance thresholds. B: The heatmap illustrates the relative expression levels of DE miRNAs. Rows represent miRNAs and columns show the samples, with a color gradient from blue (low expression) to red (high expression) indicating the z‐score normalized expression values. Hierarchical clustering was performed for both miRNAs and samples, revealing distinct expression patterns and sample grouping. [file GCC-64-e70021-s002.tif]
